# Attitudinal Acceptance of Intimate Partner Violence Among Adolescents and Young Adults in Nigeria and Tanzania: An Exploration Into Target Reference Groups

**DOI:** 10.1016/j.jadohealth.2019.10.006

**Published:** 2020-01

**Authors:** Melissa Meinhart, Ilana Seff, Gary L. Darmstadt, Ann M. Weber, Lindsay Stark

**Affiliations:** a Columbia University School of Social Work, New York, New York; b Department of Population and Family Health, Columbia University Mailman School of Public Health, New York, New York; c Department of Pediatrics, Stanford University School of Medicine, Stanford, California; d George Warren Brown School, Washington University in St. Louis, St. Louis, Missouri

**Keywords:** Intimate partner violence, Adolescents, Young adults, Violence exposure

## Abstract

**Purpose:**

Attitudinal programming for the prevention of intimate partner violence (IPV) among adolescents and young adults often focuses on *whom* to target based on gender or age; yet other pivotal junctures may relate to *when* to intervene, such as critical events (e.g., marriage). Using data from the nationally representative Violence Against Children Survey in Nigeria and Tanzania, this study examines the gendered association of acceptance of IPV across 3 reference groups—age, marital status, and education attainment—for male and female adolescents and young adults.

**Methods:**

Data were analyzed from a sample of 2,437 and 1,771 males in Nigeria and Tanzania, respectively, and 1,766 and 1,968 females in each respective country. Logistic regressions were used to estimate the odds of agreeing with at least one of 5 scenarios when it is acceptable for a husband to beat his wife. A second model examined how experience of IPV in the prior 12 months influences the attitudinal outcome for females.

**Results:**

Age was not found to be a significant predictor for attitudinal acceptance of IPV in either country or for either gender. Level of schooling was found to be a significant predictor for decreased odds of attitudinal acceptance of IPV for males but not females in both countries. In contrast, being married was associated with IPV acceptance for females in Tanzania (adjusted odds ratio [aOR]: 1.56; confidence intervals [CIs]: 1.03–2.37) and Nigeria (aOR: 1.66; CIs: 1.19–2.30), but not for males. The significance of marriage for females in Nigeria remained (aOR: 1.67; CIs: 1.20–2.33), even adjusted for past 12-month IPV experience (aOR: 1.85; CIs: 1.11–3.07) and the interaction of IPV experience and marriage (aOR: 3.42, CIs: 1.72–6.80).

**Conclusions:**

Among adolescents and young adults in Nigeria and Tanzania, there are gendered associations for attitudinal acceptance of IPV. Marriage appears to be a strong predictor for females, even adjusted for IPV experience, thus indicating that there is something unique to marriage among female adolescents and young adults that influences acceptance of IPV.

**Implications and Contribution**

This study contributes to the literature examining normative IPV attitudes within reference groups of male and female adolescents and young adults in Tanzania and Nigeria. Our findings suggest that it may be efficacious to target married females as a reference group for programming related to violence prevention for adolescents and young adults.

Intimate partner violence (IPV) is the most prevalent type of violence against women [1,2]. In addition to its inherent infringement on the Universal Declaration of Human Rights [3], IPV victimization has been correlated with negative physical and mental health outcomes across the life course [4,5]. Research also sheds light on the relationship between gendered attitudes during adolescence and young adulthood and outcomes across the life course. For example, attitudinal acceptance of patriarchal values among female adolescents—such as supporting men's perpetration of violence against women or divergent expectations for males and females—is associated with future exposure to violence [6], poor mental health [7], and adverse economic [8] outcomes for female adolescents and young adults. Research further emphasizes that attitudinal acceptance of IPV is associated with victimization for women and perpetration for men [[9], [10], [11], [12]].

Although a growing number of researchers have identified characteristics associated with attitudinal acceptance of IPV or inequitable gender roles in sub-Saharan Africa [[13], [14], [15], [16], [17]], fewer have explored how gendered IPV attitudes might emerge and solidify alongside key life events or within specific subpopulations, specifically among adolescents and young adults in sub-Saharan Africa. Adolescence and young adulthood, defined for the purpose of this study as the transformative phase between aged 10 and 24 years [18], is a key stage of life during which knowledge expands, attitudes take shape, and lifelong behaviors emerge. The innately dynamic experiences during these years create a unique opportunity for programs to effectively impact lifelong outcomes. The Gender Intensification Hypothesis posits that culturally endorsed gender roles are most strongly imposed during adolescence as a means to engrain normative behavior [19]. Research initiatives, such as the Lancet Commission on Adolescent Health [20], have also highlighted how the cascading effects of this critical period across the life course are understudied. Thus, programming specifically aimed at addressing attitudes and behaviors related to violence and gender norms is critical at this life stage, and it is important to understand how attitudes take shape to build effective programs. Although research has examined how interventions addressing IPV in sub-Saharan Africa can potentially address IPV-related attitudes [21], less is known in regards to interventions specific to adolescents in sub-Saharan Africa. Instead, attitudinal programming for adolescents often focuses on *whom* to target based on gender or age, without considering other pivotal junctures that may relate to *when* to intervene, such as critical events (e.g., marriage) or certain opportunities (e.g., educational attainment). In addition, attitudinal programming may benefit from a more nuanced understanding of how to group participants such that healthy attitudinal shifts can be mutually reinforced within relevant peer reference groups [22,23].

Understanding the structure and roles of these group dynamics is critical for practitioners working to support the well-being of adolescents and young adults, as research highlights the utility of shifting peer norms and attitudes in achieving this objective [[23], [24], [25], [26]]. In addition, communication programs that address attitudes among adolescents and young adults have demonstrated shifts in gender norms [27], and community-based interventions to promote gender-equitable attitudes have been shown to prevent IPV [28]. Although there are examples of successful programming related to attitudinal outcomes, there is a gap in empirically understanding clear pathways of attitudinal development during adolescence and young adulthood. One systematic review highlights that adolescent programming around IPV is often singularly framed around the demographic characteristic of age without consideration for the complex pathways leading to attitudinal and normative attainment [29]. A report by the International Rescue Committee echoes this sentiment and recognizes how improved targeting of participants, beyond overt demographic and displacement factors, could further improve the outcomes of its current women's protection and empowerment programming [30].

Although existing research stresses the importance of engaging adolescents and young adults in programming related to gender dynamics, violence, and health, there are gaps in the literature with respect to when to intervene during this life stage for optimum outcomes. This study seeks to understand when and with whom gendered attitudes seem to solidify in adolescents and young adults, through examining attitudinal acceptance of IPV among females and males aged 13–24 years in Nigeria and Tanzania. The analysis further explores how experiences of IPV in the prior 12 months influence attitudinal acceptance of IPV for females. The nationally representative nature of the analysis further allows researchers, practitioners, and policymakers to begin to understand what is normative in a given group context. Specifically, the study adds to the literature by exploring the extent to which age, education, and marriage influence attitudinal acceptance of IPV.

## Methods

### Study setting

This study included data from 2 countries from sub-Saharan Africa: Nigeria and Tanzania. Moving beyond geographical proximity, there are important linkages between these settings. Although Nigeria boasts Africa's largest economy, each country is classified as a low- or middle-income country with the 2018 per capita GDP at $5,980 in Nigeria and $3,227 in Tanzania [31]. Also on the macrolevel, both countries have strong commitments to addressing violence against children. Nigeria instituted the “Year of Action to End Violence against Children” [32], and Tanzania implemented the “National Plan of Action to Prevent and Respond to Violence against Children” [33]. There are also important demographic similarities. For example, the median age of marriage for females is similar in both countries (18.1 years in Nigeria and 19.2 years in Tanzania) [34,35]. The age of first marriage is higher for males in both countries, with the median age in Nigeria as 27.2 years and the median age in Tanzania as 24.3 years. Although there are cultural, political, and geographical differences between these countries, we believe expanding the frame of focus beyond a single country can inform broader considerations for responses across sub-Saharan Africa. Furthermore, the nationally representative nature of Violence Against Children Survey (VACS) data from these 2 countries can inform the generalizability of study findings.

### Study participants and procedures

Data for this study were drawn from the publicly available VACS in Tanzania and Nigeria; data were collected in 2009 [36] and 2014 [37], respectively. The VACS are part of the Together for Girls partnership led by the U.S. Centers for Disease Control and Prevention. The cross-sectional household surveys provided information on exposure to sexual, physical, and emotional violence among females and males, aged 13–24. Questionnaires also included questions on the circumstances surrounding violence, potential protective factors and consequences of violence, and attitudes related to gender inequity and IPV. The VACS are nationally representative and used a three-stage, stratified, multistage sampling approach [38]. In the first stage, 100 and 353 primary sampling units or enumeration areas (EAs) were selected in Tanzania and Nigeria, respectively. In the second stage, approximately 20 and 30 households, respectively, were selected from each EA. Finally, in the third stage, one eligible household member was randomly selected for inclusion in the sample. Data collection yielded a sample of 2,437 and 1,771 males in Nigeria and Tanzania, respectively, and 1,766 and 1,968 females in each respective country. In addition, the VACS used a “split-sample” strategy, whereby each EA comprised either all males or all females. This sampling protocol was used to minimize the likelihood that both a survivor and perpetrator of the same incident of violence were interviewed, thus minimizing risk to the survivor. VACS included randomly selected participants, whereby the heads of household in each survey gave consent for the survey and provided basic socioeconomic information on the household. Verbal consent was then provided by all participants aged 18 years or older, and verbal assent was provided by the head of household for respondents aged younger than 18 years. Data were collected through in-person interviews by same-sex interviewers (i.e., males with males and females with females). Surveys were conducted in a quiet and private space whenever possible. All data were captured on a laptop or netbook through Electronic Data Capture using CSPro. Data collection took approximately 1–2 months in each country.

The VACS studies were approved through the Centers for Disease Control and Prevention's Institutional Review Board and an in-country ethics review board. Given the sensitive nature of the survey, each country had its own referral plan to appropriately respond to respondents’ needs.

### Measures

#### Independent variables

This study examined the associations between attitudinal acceptance of IPV with 4 independent variables: age, marital status, educational attainment, and experiencing IPV in the last 12 months. All variables were self-reported. Age was measured as a continuous variable. For marital status, respondents were assigned a “1” if they had ever been married or lived with someone as if married and a “0” if not. There were slight differences in the operationalization of educational attainment between countries, given small differences in the survey questionnaires. In Tanzania, the measure of educational attainment comprised 4 categories, including never attended school, attended at least primary school, attended at least secondary school, and attended more than secondary school; in Nigeria, the 5 category options consisted of: never attended school, attended but did not complete primary school, completed primary school, completed secondary school, and completed more than secondary school. Finally, female respondents were identified as having experienced IPV in the last 12 months if the most recent or first experience of physical or sexual IPV occurred in the last 12 months.

#### Outcome of interest

The outcome of interest for this study was a measure of attitudinal acceptance of IPV. Our measure was constructed using 5 binary survey questions assessing when the respondent believes it is acceptable for a man to beat his wife. Respondents were provided a prompt—“*Occasionally, a man may be angered by his wife's actions. Do you think a man has the right to beat his wife if:*”—then asked to indicate whether they agreed with each of the following 5 statements: “*If she goes out without telling him*”; “*She neglects the children*”; “*She argues with him*”; “*She refuses to have sex with him*”; and “*She burns the food.*” For each statement, respondents were assigned a “1” if they agreed and a “0” if they disagreed. As our intention was to measure the attitudinal acceptance of IPV under any circumstance, the final measure was created by indicating whether a participant agreed with at least one of the statements supporting IPV, such that a value of “1” reflects attitudinal acceptance of IPV and a value of “0” attitudinal disapproval of IPV [[14], [15], [16],^39^].

### Statistical analysis

All analyses were implemented using Stata 14 [40]. Logistic regression models first assessed the associations between age, marriage, and education attainment and the key outcome of interest: agreeing with at least one scenario in which it is acceptable for a man to beat his wife. Next, to help elucidate any potential pathway between marital status and acceptance of IPV, the models above were re-estimated with an additional covariate capturing IPV victimization in the last 12 months and its interaction with marital status. Analyses were implemented separately for each country and were stratified by sex. Standard errors were adjusted for the complex sampling design, and all observations were weighted to be representative of the population of adolescents and young adults, aged 13–24 years, in each country.

Because of the sensitive nature of the survey questions underlying the outcome of interest and potential social desirability bias, data were missing for the outcome for approximately 9% in Tanzania and 10% in Nigeria. Chi-squared tests were used to determine whether missingness of the outcome was correlated with the 3 predictors included in the model. We also implemented 2 sensitivity analyses by first conducting our main analysis on imputed data, then replicating our main analysis using Demographic and Health Survey (DHS) data from Nigeria [35] and Tanzania [34]. For the first set of sensitivity analyses, we used a multiple imputation method, using “mi impute” in Stata to generate a set of 5 imputations. For the second set of sensitivity analyses, we used a sample of males and females between the ages of 15–25 years from the 2013 and 2015–2016 DHS data in Nigeria and Tanzania, respectively. Using the DHS sample, we replicated the main model of logistic regressions to assess the relationship between age, marriage, and school attendance and the key outcome of interest: agreeing with at least one scenario in which it is acceptable for a man to beat his wife. The 5 binary survey questions—assessing when the respondent believes it is acceptable for a man to beat his wife—are the same in the DHS and VACS, and most of the independent variables were operationalized identically in both datasets. The one exception was educational attainment, whereby the schooling categories in the DHS were “no schooling,” “incomplete primary,” “complete primary,” “incomplete secondary,” “complete secondary,” or “completed more than secondary.”

## Results

Table 1 summarizes the descriptive statistics of the samples. The average sample age for both males and females, and in both countries, was approximately 18 years. Females in both countries were significantly more likely than males to be married or living with someone as if married (30.2% vs. 10.9% in Nigeria and 25.7% vs. 7.2% in Tanzania). In Nigeria, females were twice as likely to report having experienced IPV in the last 12 months compared with males (7.9% vs. 3.5%; *p* < .001). In contrast, in Tanzania, approximately 8%–9% of both males and females reported IPV exposure. While only significant in Nigeria (*p* < .001), females in both countries were more likely to agree with at least one IPV statement than males in the respective country (37.7% vs. 28.2% in Nigeria and 55.4% vs. 49.2% in Tanzania). Attitudinal acceptance of IPV was more common in Tanzania than in Nigeria.

Table 2 summarizes VACS findings regarding attitudinal acceptance of IPV. Age was not found to be a significant predictor for attitudinal acceptance of IPV in either country or for either gender. Being married was consistently associated with IPV acceptance for females in both countries, but not for males. Controlling for age and schooling, married females in Nigeria and Tanzania had 1.66 (*p* = .003; confidence intervals [CIs]: 1.19–2.30) and 1.56 (*p* = .035; CIs: 1.03–2.37) greater odds, respectively, of agreeing with at least one statement supporting IPV compared with their unmarried counterparts. In contrast, level of schooling was found to be a significant predictor for decreased odds of attitudinal acceptance of IPV for males but not females in both countries. Specifically, compared with Nigerian males with no schooling, those who had completed secondary or tertiary school had lower odds of agreeing IPV was acceptable (adjusted odds ratio [aOR]: .51; *p* = .023; CIs: .29–.91; and aOR: .19; *p* = .002; CIs: .07–.52, respectively); similar patterns were observed for schooling status among males in Tanzania. The sensitivity analyses with DHS data yielded similar findings between marriage and attitudinal acceptance of IPV, whereby married females in Nigeria (aOR: 1.11, *p* < .05; CIs: 1.01–1.21) and Tanzania (aOR: 1.22, *p* < .01; CIs: 1.06–1.40) had significantly greater odds of agreeing with at least one statement supporting IPV compared with unmarried females. The analyses from the DHS data corroborated that there were no significant associations between marriage and IPV attitudes for males in either country.

**Table 1:** Descriptive statistics among adolescents and young adults aged 13–24 years

	Nigeria			Tanzania			Across-country *p* value	
	Males	Females	*p*-value	Males	Females	*p*-value	Males	Females
Age	18.2 (3.5)	18.4 (3.6)	.07	17.9 (3.3)	18.2 (3.4)	.163	.163	.201
Married	10.9	30.2	<.001	7.2	25.7	<.001	.036	.163
Ever attended school			<.001			<.001		
No school	13.0	22.5		5.3	10.1			
Attended primary, but did not complete (Nigeria)/attended at least primary (Tanzania)	9.2	8.6		60.6	63.9			
Completed primary (Nigeria)/attended at least secondary (Tanzania)	46.2	35.0		33.2	25.3			
Completed secondary (Nigeria)/attended more than secondary (Tanzania)	29.1	31.5		.9	.8			
Completed more than secondary	2.5	.1						
Experienced IPV, last12 months	3.5	7.9	<.001	8.6	8.4	.088	.003	.055
Agree with at least oneIPV statement	28.2	37.7	<.001	49.2	55.4	.104	<.001	<.001
Sample size (n)	2,437	1,766		1,771	1,968			

Data are (%) or mean (SD). Marriage is defined as being in a formal marriage or living with someone as if married. All observations are weighted to be representative of the population in each country. The differences between males and females were assessed using adjusted Wald tests.

IPV = intimate partner violence.

**Table 2:** Predicting attitudinal acceptance of IPV, adjusted odds ratios (aOR)

	Males aOR (95% CI)	Females, aOR (95% CI)
Nigeria		
Age	1.03 (.99–1.06)	1.00 (.96–1.04)
Marriage	.9 (.63–1.28)	1.66** (1.19–2.30)
Schooling level (reference group = no schooling)		
Attended primary, but did not complete	.81 (.38–1.75)	.62 (.31–1.22)
Completed primary	.78 (.44–1.39)	1.23(.83–1.82)
Completed secondary	.51* (.29–.91)	.72 (.44–1.16)
Completed more than secondary	.19** (.07–.52)	.67 (.25–1.76)
Tanzania		
Age	.97 (.92–1.03)	1.06 (1.00–1.13)
Marriage	.78 (.38–1.62)	1.56* (1.03–2.37)
Schooling level (reference group = no schooling)		
Attended at least primary	1.32 (.93–1.86)	1.75 (.90–3.39)
Attended at least secondary	.60* (.38–.94)	1.37 (.64–2.93)
Attended more than secondary	.09* (.01–.60)	.27 (.02–3.04)

Logistic regressions are used to estimate the odds of agreeing with at least one scenario in which it is acceptable for a husband to beat his wife. Marriage is defined as being in a formal marriage or living with someone as if married. Standard errors are adjusted for complex sampling design. All observations are weighted to be representative of the population. Odds ratios are significant at *p < .05 and **p < .01.

CI = confidence interval; IPV = intimate partner violence.

Table 3 presents female results from the same models presented in Table 2, but with IPV exposure added as a covariate and interaction term with marital status. In Nigeria, being married and reporting exposure to IPV in the last 12 months were both independently associated with IPV acceptance (aOR: 1.67; *p* = .003; CIs: 1.20–2.33; and aOR: 1.85; *p* = .18; CIs: 1.11–3.07, respectively). Furthermore, females in Nigeria who were both married and reported experiencing IPV were significantly more likely to express acceptance of IPV (aOR: 3.42; *p* = .001; CIs: 1.72–6.80) compared with females who were unmarried and had not experienced IPV. In Tanzania, exposure to IPV in the last 12 months was not found to be statistically significantly associated with attitudinal acceptance of IPV; and, furthermore, marriage was no longer predictive of the outcome when including IPV exposure in the model. All findings were robust to sensitivity analyses using imputed data.

**Table 3:** Predicting attitudinal acceptance of IPV for females, adjusted odds ratios (aOR)

	Nigeria aOR (95% CI)	Tanzania aOR (95% CI)
Age	.99 (.95–1.03)	1.06 (1.00–1.13)
Marriage	1.67** (1.20–2.33)	1.44 (.97–2.14)
Experienced IPV, last 12 months	1.85* (1.11–3.07)	1.61 (.33–7.83)
Marriage × experienced IPV, last 12 months	3.42** (1.72–6.80)	2.60 (.99–6.86)
Schooling level (reference group = no schooling)		
Attended primary, but did not complete (Nigeria)/attended at least primary (Tanzania)	.62 (.31–1.22)	1.76 (.89–3.49)
Completed primary (Nigeria)/attended at least secondary (Tanzania)	1.22 (.83–1.80)	1.38 (.63–3.02)
Completed secondary (Nigeria)/attended more than secondary (Tanzania)	.7 (.43–1.13)	.29 (.03–3.32)
Completed more than secondary	.62 (.28–1.09)	

Marriage is defined as being in a formal marriage or living with someone as if married. Standard errors are adjusted for complex sampling design. All observations are weighted to be representative of the population. Odds ratios are significant at *p < .05 and **p < .01.

CI = confidence interval; IPV = intimate partner violence.

## Discussion

This study adds to the existing literature by demonstrating that critical life experiences may shape attitudes around IPV for adolescents and young adults. Although the Gender Intensification Hypothesis proposes a linkage between age and shaping attitudes or norms, our study highlights that experiences or events (such as marriage) may be influential in predicting acceptance of IPV. Most notably, our findings highlight that married girls were more likely to exhibit attitudinal acceptance of IPV in Nigeria and Tanzania, compared with their unmarried peers.

Age may have presented as a proxy for life experiences or events in previous research. By adding marriage and schooling into an adjusted model, there was no emerging significance of age. Instead, our findings suggest that marriage may have a unique association with IPV-supportive attitudes for female adolescents and young adults, even after controlling for experiencing IPV. Future research could usefully explore if and how marriage influences attitudes around IPV, specifically for young women, as well as explore how other experiences may influence IPV attitudes. Girls who have entered into marriage may seek advice from one another [26] and endorse tolerating violence to keep families together or to align with perceived social norms [41]. Alternatively, it is not unreasonable to hypothesize that female adolescents and young adults who believe IPV is acceptable are themselves more likely to get married in adolescence and early adulthood if there is an implicit understanding that IPV is likely to occur during a marriage. Importantly, regardless of the directionality of the relationship between marriage and attitudinal acceptance of IPV in these settings, these attitudes are stronger (and likely continually reinforced) among married, compared with unmarried, young women.

Social norms theory argues that proper identification of relevant reference groups is critical for effectively measuring and targeting changes in norms [42]. Empirical evidence suggests that measuring attitudes within and across groups can help reveal the boundaries of key reference groups and the norms within [[42], [43], [44], [45]]. Our analysis underscores the importance of moving beyond gender and age as assumed reference groups for attitudinal programming and suggests that targeted programming may support young, married girls in Tanzania and Nigeria. As such, programming that aims to reduce acceptance of IPV among female adolescents and young adults may need to account for differences in attitudes and interactions between married and unmarried girls. These findings further highlight the need for early education around healthy relationships, for both married and unmarried girls. Even if specific marriage-based reference groups are not created for programming, considerations around marriage can be made. For instance, programs that have group activities or didactic sessions should consider how married and unmarried girls are distributed across program groups.

A few limitations should be taken into consideration when reviewing this study. Although findings should not be generalized to all of Sub-Saharan Africa, these data are nationally representative for the 2 countries included in the analysis—Nigeria and Tanzania—and may inform the development of future studies in Sub-Saharan Africa. Furthermore, the cross-sectional nature of these data impedes temporal and, thus, causal interpretation. These data also did not allow for clustering or geolocation of the reference group bounds for a given adolescent nor did they facilitate the study of the association between married and unmarried girls within the same communities. Thus, the reference groups used in our analysis do not necessarily relate to localized influence. Instead, the robust nature of these results, including generalizability to different samples (i.e., DHS), allowed for a critical exploration into the role of reference groups on attitudes toward IPV for both female and male adolescents and young adults, as well as urging for increased research surrounding this topic.

The influence of reference groups on attitudes, behaviors, and norms is critically important during the age of adolescence and young adulthood. In addition to including considerations of age and gender in programming, unique opportunities for intervention may relate to the critical juncture of marriage, especially for female adolescents or young adults. As attitudes solidify, the potentially self-perpetuating norms in favor of IPV in married females can carry lifelong effects. Our research highlights how the critical juncture of marriage may also influence attitudes toward IPV and offers insight into how programming may use reference groups to tailor attitudinal programming related to IPV in sub-Saharan Africa for adolescents and young adults.
