# Erratum

**DOI:** 10.1016/j.jadohealth.2019.12.015

**Published:** 2020-03

In Meinhart M, Seff I, Darmstadt GL, Weber AM. Attitudinal Acceptance of Intimate Partner Violence Among Adolescents and Young Adults in Nigeria and Tanzania: An Exploration Into Target Reference Groups Order and Affiliation of Authorship. J Adolesc Health 2020;66:S3–S8. https://doi.org/10.1016/j.jadohealth.2019.10.006.

The title is incorrect. The correct title is, “Attitudinal Acceptance of Intimate Partner Violence Among Adolescents and Young Adults in Nigeria and Tanzania: An Exploration Into Target Reference Groups.”

